# Differences in the Quantity and Types of Foods and Beverages Consumed by Canadians between 2004 and 2015

**DOI:** 10.3390/nu11030526

**Published:** 2019-02-28

**Authors:** Claire N. Tugault-Lafleur, Jennifer L. Black

**Affiliations:** Food, Nutrition and Health Program, 2205 East Mall, The University of British Columbia, Vancouver, BC V6T 1Z4, Canada; j.black@ubc.ca

**Keywords:** diet surveys, food groups, secular trends, Canada

## Abstract

This study examined differences in food and beverage intake estimated from nationally representative surveys of Canadians in 2004 and 2015 collected through the Canadian Community Health Surveys. Differences in mean daily energy intake and amounts of food consumed were compared between 2004 and 2015 and across age groups for all energy reporters (aged 2 years+) and among only plausible energy reporters. From 2004 to 2015, mean energy intake decreased by 228 kcal/day (all energy reporters) and 74 kcal/day (plausible energy reporters). Canadians reported consuming more daily servings of meat and alternatives but fewer servings of vegetables and fruit and milk and alternatives in 2015 compared to 2004. Analyses of food subgroups revealed that Canadians reported consuming more daily servings of dark green and orange vegetables, dairy products, legumes, nuts and seeds, and eggs but fewer servings of potatoes, other vegetables, fruit juices, fluid milk, and sugar-sweetened beverages in 2015 compared to 2004. While some aspects of the Canadian diet have improved, daily mean intake of other nutritious foods either stagnated or worsened over time. Continued attention is needed to improve population-level intakes of vegetables, fruit, whole grains, and protein foods such as legumes, nuts, seeds, and lower fat dairy products.

## 1. Introduction

In Canada, poor dietary quality is a leading contributor to the burden of chronic diseases, including type 2 diabetes, cardiovascular diseases, and some cancers [1]. Moreover, the economic cost associated with not meeting national dietary recommendations is estimated to contribute substantially to the Canadian health care system [2]. Analyses of nationally representative dietary data from the 2004 Canadian Community Health Survey (CCHS) suggest that large gaps exist between reported dietary intakes and national recommendations for healthy eating. For example, in 2004, only 26% of the population aged 2 years and older met the minimum number of daily servings of vegetables and fruit recommended by Eating Well with Canada’s Food Guide (referred to here as the 2007 CFG) for their respective age–sex group [3]. Meanwhile, approximately 22% of total daily calories consumed by Canadians aged 4 years and older in 2004 came from minimally nutritious foods, such as high fat and/or high sugar foods and sugar-sweetened beverages [4].

Between 2007–early 2019, national dietary recommendations advocated by the 2007 CFG (the food guide in use during the time of this study’s writing) [5] included specific recommendations regarding amounts and types of foods to consume within each of four core food groups: Vegetables and fruit, grain products, milk and alternatives, and meat and alternatives [6]. Within a food group (e.g., vegetables and fruit), foods can vary in their nutritional content. Therefore, the 2007 CFG included statements on the quality of food choices within each food group to promote nutrient adequacy within a food intake pattern [6]. For example, the statement “Have vegetables and fruit more often than juice” was issued to maintain the fibre content of the food intake pattern, while the statement “Eat at least one dark green and one orange vegetable each day” aimed at achieving adequate levels of dietary folate and vitamin A [6]. Within grain products, the 2007 CFG emphasized the consumption of whole grains over non-whole grains to achieve optimal intakes of magnesium and fibre [6]. The guidance recommending daily consumption of low fat milk (skim, 1% or 2%) was based on “the effectiveness of obtaining adequate calcium and vitamin D while remaining within an appropriate macronutrient profile and total amount of calories” within a food intake pattern [6]. The Canadian dietary guidelines have recently undergone major revisions [7] and understanding whether Canadians have changed the average quantity and composition of food choices over time can provide foundational knowledge to help to inform future research and public health practice aimed at improving national nutritional outcomes.

National-level analyses based on dietary data from the 2004 CCHS have previously shown differences in food group intake according to age group. In 2004, 7 out of 10 children aged 4–8 years reportedly consumed less than 5 servings of vegetables and fruit, whereas around half of adults fell short of the 1992 CFG recommended minimum of 5 servings a day (the recommendations in place at the time of the 2004 CCHS) [4]. Black and Billette showed that in 2004, usual intakes of fruit juices were higher among children and adolescents compared to adults, while adults consumed higher usual intakes of dark green vegetables compared to children [3]. In 2004, Canadian adolescents had, on average, the highest proportion of their total daily calories (25%) obtained from minimally nutritious foods compared to other age groups [4]. Whether food intake reported in 2004 across age groups has changed in more recent years remains unknown.

In 2015, Statistics Canada carried out another nationally representative dietary survey (the CCHS-2015 Nutrition), with a population-representative sample and dietary assessment methods similar to the 2004 CCHS [8]. Recent analyses have examined differences over time in energy and macronutrient intakes reported by Canadian children and adults [9,10,11]. Still, little published work to date has described differences over time in Canadians’ average intakes of foods and beverages. Therefore, the purpose of this study was to examine differences in the quantity and types of foods and beverages reported by Canadians aged 2 years and older from 2004 to 2015. A secondary objective was to test whether dietary differences reported between 2004 and 2015 varied across age groups.

## 2. Materials and Methods

### 2.1. Data Source and Analytical Sample

Nationally representative data were obtained from the 2004 and 2015 CCHS. Both surveys used a multistage stratified cluster sample that was nationally representative for age, sex, geography, and socioeconomic status (*n* = 35,107; response rate, 76.5% in 2004 [12,13]; *n* = 20,487; response rate 61.6% in 2015 [8]). The surveys targeted residents of all ages (in 2004) and ages 1 year and above (in 2015) living in private dwellings in Canada’s ten provinces [8]. The target population did not include full-time members of the Canadian Forces or Canadians living in the Territories, on reserves or other Aboriginal settlements, or institutionalized populations (e.g., persons living in prisons or care facilities) [14]. 

**Figure 1:**
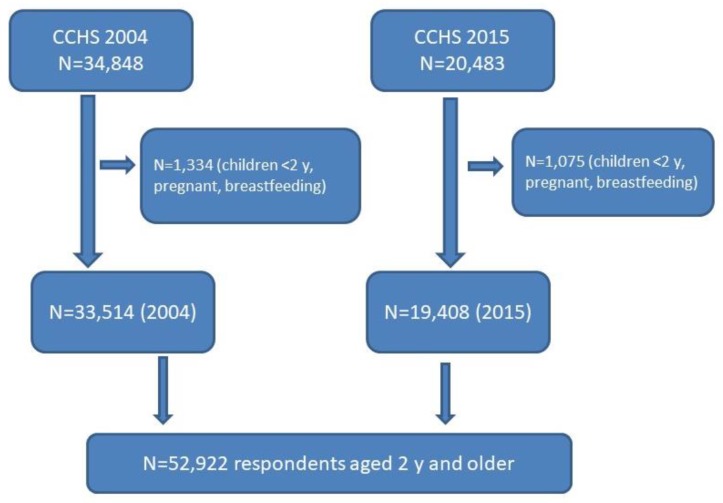
Analytical sample.

In both 2004 and 2015, trained interviewers used a computer-assisted 24-h dietary recall, asking respondents about foods and beverages consumed in the 24-h period during the day prior to the interview—including types and amounts of foods consumed, eating occasion (e.g., breakfast, lunch, snack), and time of consumption [8,12] This method was adapted from the United States Department of Agriculture Automated Multiple-Pass Method [15] and uses an automated questionnaire that guides the interviewer through a series of questions and probes to maximize the interviewees’ opportunities for remembering and reporting foods eaten. Interviews for children age 5 years or younger were conducted with the parents or caregivers, and interviews with children age 6 to 11 years were conducted with parental assistance. Children aged 12 years and above were asked to provide their own information. The present study used the first interviewer-administered 24-h recall for both survey years (2004 and 2015), since the mean of one-day intakes is an acceptable estimate of the mean ‘usual’, or long-term daily average intake of a population when it is properly estimated; that is, when the days of the week and seasons of the year are adequately represented [16], which was the case for both the 2004 [12] and 2015 [8] waves of the CCHS. Figure 1 shows the process used for deriving the analytical sample of this study. The sample included respondents aged 2 years and older, who were not pregnant or breastfeeding. Pregnant and breastfeeding women were excluded because insufficient data were available with which to calculate their total energy expenditure (TEE), which is required to assess potential misreporting of energy (see ‘Energy Misreporting’ section below).

The final analytical sample included 33,514 and 19,408 respondents in 2004 and 2015, respectively. The ethical approval for population surveys conducted by Statistics Canada, such as the 2004 and 2015 CCHS-Nutrition, is based on the authority of the Statistics Act of Canada. Access to the CCHS data files was provided by the Statistics Canada Research Data Centre Program [17].

### 2.2. Food Groupings

The Canadian Nutrient File (CNF) is Canada’s standard reference food composition database used in the 2004 and 2015 CCHS [8,12]. In 2014, Health Canada released the Health Canada Surveillance Tool system (HCST), a nutrient profiling system which classifies foods and beverages in the CNF according to how closely they aligned with the 2007 CFG guidance [18]. The HCST has been previously described [18,19]. Briefly, foods in the CNF are classified into one of the four core food groups from the 2007 CFG (vegetables and fruit, grain products, milk and alternatives, meat and alternatives). Foods in the CNF are also assigned codes for specific food subgroups (e.g., dark green vegetables, fruit juices, whole grains, fluid milk) based on their nutrient content, similarities, and alignment with the 2007 CFG guidance around types of foods to choose within each of the four food groups [18]. Within this framework, foods are also classified as falling within one of four tiers—where Tier 1 foods are considered the most nutritious and Tier 4 the least nutritious foods. This analysis drew on amounts contributed by all four tiers together (Tier 1–4) since Tier 4 foods technically belonged to the four core food groups from the 2007 CFG. A detailed description of the food group and food subgroups used for these analyses is presented in Table 1. The food groupings used in this study were adapted from the original HCST classification system [18], with some slight modifications to reduce the number of dietary intake variables and to combine nutritionally similar food subgroups—for instance, the subgroups of fish and shellfish were combined here. For the core four food groups (e.g., vegetables and fruit) and food subgroups (e.g., whole fruit) in the 2007 CFG, we measured intake using servings [18].

The 2007 CFG also included recommendations around other foods (foods and beverages that were not part of the four major food groups). These other foods included minimally nutritious foods (i.e., high fat and/or high sugar foods such as candies, chocolate, sauces) and other beverages. Within the HCST framework, other beverages are further classified into high-calorie beverages providing more than 40 kcal/100 g serving (e.g., sugar-sweetened beverages such as non-diet sodas, energy drinks), low-calorie beverages providing 40 kcal/100 g serving (or less) (e.g., diet sodas, unsweetened or lightly sweetened tea or coffee), and alcohol-containing beverages (e.g., wine, beer). For other foods and other beverages, these analyses considered energy intake (kcal) from these foods, since there were no standard reference amounts (servings) for these foods [18].

### 2.3. Sample Characteristics

Changes in sample demographic and lifestyle characteristics (e.g., increased proportion of immigrants, an aging population, fewer smokers) may lead to changes in reported intakes of types or quantities of foods. Several sociodemographic and lifestyle variables were reported in the CCHS general health questionnaire and compared between 2004 and 2015. These variables were considered as potentially confounding factors when assessing associations between time and dietary intakes. Continuous variables included: Age (in years) and body mass index (BMI) (kg/m^2^) (for respondents aged 18 years and above). Similar to the approach used by Barr et al. [20], age group was divided into five categories representing key life course stages: Young children (2–5 years), children (6–12 years), adolescents (13–17 years), adults (18–54 years), and older adults (≥55 years). Other categorical variables were dichotomized as follows: Sex (male vs. female), location of residence (urban vs. rural), immigrant status (immigrant to Canada vs. born in Canada), ethnicity (White/Caucasian vs. non-White), household-level education (university graduate vs. lower educational attainment), smoking status (smoker vs. past/never smoked) (for respondents aged 12 years and older), supplement use (any use of a nutritional supplement in the past 30 days vs. no use), and overweight/obesity (for respondents aged 2–17 years, this was based on body mass index (BMI) z-scores for age and sex; for respondents aged 18 years and older, this was defined as a BMI >25 kg/m^2^).

### 2.4. Energy Misreporting

Self-reported dietary intake is subject to misreporting (i.e., respondents over- or underestimate their dietary intake) [21]. Changes to misreporting over time (such as an increase in energy underreporting from 2004 to 2015) could pose problems when attempting to compare dietary intakes because underreporting (not true differences in intake) could fallaciously contribute to estimated differences in dietary intakes [11]. Using a comparable energy reporting category of respondents (e.g., only plausible energy reporters in 2004 and 2015) has been proposed as one potential solution to improve the quality of comparisons when examining dietary intakes over time [11].

In these analyses, we classified respondents as either plausible, under- or over-energy reporters using the Institute of Medicine TEE equations [22] in a subsample of respondents with measured heights and weights (*n* = 20,738 in 2004 and *n* = 13,611 in 2015). We used the method developed by McCrory and colleagues [23], which had been previously used with the 2004 CCHS [21]. Physical activity was assessed differently in 2004 and 2015, precluding the use of physical activity estimates from questions in the two surveys. Instead, fixed levels of physical activity (by age group) were assumed for the entire population [11]. Children younger than 14 years of age were presumed to be low active, while adolescents aged 14 years or older and adults were assumed to be sedentary. These levels are consistent with the average physical activity levels measured directly among Canadian children and adolescents from 2007 to 2015 [24]. Respondents were classified based on the percentage of their TEE that they reported as energy intake: Less than 70%, underreporters; between 70% and 142%, plausible reporters; and over 142%, overreporters.

In line with previous findings reporting changes to energy misreporting over time [11], the proportion of energy underreporters increased from 22% to 30%, while the proportion of energy overreporters decreased from 17% to 9% (Table 2). Therefore, to evaluate the potential impact of energy misreporting on the differences in food group intakes over time, results generated from analyses including all energy reporters (under-, plausible and over-energy reporters combined) were compared to results generated from analyses which included only plausible energy reporters.

### 2.5. Statistical Analyses

Differences in sample characteristics between survey years were compared using Rao–Scott Chi square tests (for categorical variables such as energy misreporting, urban vs. rural area of residence) and two-sample *t* tests (for continuous variables such as age (years) and BMI (kg/m^2^)). Simple linear regression models were first used to compare differences in dietary outcomes (e.g., daily energy intake, servings of vegetables and fruit) between 2004 and 2015 for all energy reporters (under-, plausible and over-energy reporters combined), and among only plausible energy reporters. Second, multivariable linear regression models were used to adjust for the potentially confounding effects of total daily energy intake and sociodemographic and lifestyle variables (age, ethnicity, immigration status, household-level education, supplement use, smoking status), which differed between the 2004 and 2015 population samples. Differences in mean daily amounts of food groups and food subgroups in 2004 and 2015 were computed for all age groups combined (i.e., Canadians aged 2 years and older) as well as for each age group separately. To determine whether age group moderated any changes in food group intakes over time (for example, whether the change in vegetables and fruit intakes differed by age group), Wald tests for the joint significance of the interaction product terms were used. Finally, to express the magnitude of the change relative to food group intakes in 2004, we computed the relative percent differences in food group intakes in 2015 relative to food group intakes in 2004. This was calculated by taking the estimated mean intake difference (in servings or kcal) between survey years (all age groups combined), divided by the intake reported in 2004. The difference was then multiplied by 100 to express this difference in relative (%) terms. Relative percent differences in food group intakes were computed both for all energy reporters as well as among only plausible energy reporters.

All analyses applied the survey sampling weights to account for unequal probabilities of selection due to the complex sampling design and applied the “pooled approach” to survey weights described by Thomas and Wannell [25]. Robust estimates of sampling variance were obtained using the bootstrap method to weighted resampling, with the 500 sets of replication weights supplied by Statistics Canada. Missing data were handled with case-wise deletion, except for weight status and smoking, where dummy variables were created to account for a larger proportion of respondents with missing data (“refusals”, “don’t know”, “not stated” or “not applicable”). For example, 12,776 respondents in 2004 did not have their weight and height measured (~38% of the sample), so it was not possible to calculate a BMI (and hence, assign a weight status category). In 2015, 5797 respondents (~30% of the sample) did not have their weight and height measured. The variable pertaining to smoking was not asked to children <12 years [26,27]. Therefore, a higher proportion of the sample of Canadians aged 2 years and older were marked as ‘missing’ (7219 respondents in 2004 or ~22% of the sample; 3447 respondents in 2015 or ~18% of the sample). Database management, coding, and statistical analyses were conducted using Stata 13 (LP Stata Corp, College Station, Texas, USA), with statistical significance defined as *p*-value ≤ 0.05.

## 3. Results

### 3.1. Sociodemographic Characteristics: 2004 vs. 2015

Table 3 presents the sociodemographic and lifestyle characteristics of Canadians aged 2 years and older in 2004 and 2015. Consistent with previously reported trends in changing sociodemographic characteristics [28], the average age of the Canadian population significantly increased by ~3 years from 2004 to 2015. From 2004 to 2015, the proportion of Canadians self-identifying as White/Caucasian significantly decreased. Consistent with previous national-level analyses reporting similar trends [28], the proportion of immigrants and Canadians living in households where at least one member graduated from university significantly increased. In 2015, the average BMI among adults significantly increased (by 0.4 kg/m^2^), but the proportion of overweight/obese adults (with BMI ≥ 25 kg/m^2^) or children (defined as children with a BMI Z-score over the 85th percentile for their age and sex) did not change over time. The proportion of smokers significantly decreased (by ~6 percentage points), while the proportion of Canadians reporting the use of a supplement in the past 30 days significantly increased (by ~5 percentage points).

### 3.2. Energy Intake of Canadians by Energy Reporting Status: 2004 vs. 2015

Table 4 compares the mean total daily energy intake reported by Canadians aged 2 years and older in 2004 and 2015 for all energy reporters (left-hand side of the table) and among only plausible energy reporters (right-hand side of the table). Average daily intake from all food sources significantly decreased from 2004 to 2015 (by 228 kcal/day and 74 kcal/day for all energy reporters and among only plausible energy reporters, respectively). Average daily energy intake decreased significantly for all age groups except for older adults in analyses including only plausible energy reporters.

### 3.3. Mean Daily Intakes from the Four 2007 CFG Foods Groups: 2004 vs. 2015

Table 5 compares the covariate-adjusted average servings of food groups (derived from the 2007 CFG) and food subgroups for all energy reporters (left-hand side of the table) and for only plausible energy reporters (right-hand side of the table) in 2004 and 2015. 

## 4. Vegetables and Fruit

In 2004, Canadians aged 2 years and older reported consuming, on average, ~5.2 daily servings of total vegetables and fruit. From 2004 to 2015, the amount of daily servings of total vegetables and fruit decreased significantly, both among all energy reporters (−0.7 servings/day) and well as in analyses restricted to only plausible energy reporters (−0.6 servings/day). In 2004, the largest contributor to total vegetables and fruit were other vegetables (i.e., non-dark green and orange vegetables such as cucumbers, tomatoes, celery, corn), which represented ~33% of total daily servings of vegetables and fruit. In 2015, the largest contributors to total vegetables and fruit were both other vegetables and whole fruit (each representing ~28% of all daily servings of vegetables and fruit). 

Among all energy reporters (all ages combined), Canadians reported consuming significantly more average daily servings of dark green and orange vegetables (+0.1 servings/day) but fewer other vegetables (−0.4 servings/day), potatoes (−0.1 servings/day), and fruit juices (−0.2 servings/day). Statistically significant differences for other vegetables, potatoes, and fruit juices were found in analyses restricted to plausible energy reporters. In both survey years, average intakes of dark green and orange vegetables and other vegetables were highest among adults and lowest among adolescents and children. Average intake of fruit juices was highest among children and adolescents and lowest among older adults in both 2004 and 2015.

The magnitude of the reduction in total vegetables and fruit varied by age group (*p*-value for the overall interaction <0.001). Adolescents, adults, and older adults reported, on average, significantly fewer daily servings of total vegetables and fruit in 2015 than in 2004, whereas children reported no difference over time. The changes in daily intakes of other vegetables, whole fruit, and fruit juice also varied by age groups (*p*-values for the overall interactions <0.05). For example, adolescents, adults and older adults (all energy reporters) reported significantly fewer average daily servings of other vegetables in 2015 compared to 2004, whereas children (all energy reporters) reported no difference over time. Although children (all energy reporters) and adolescents (all energy reporters and only plausible energy reporters) reported, on average, significantly more daily servings of whole fruit in 2015 compared to 2004, no difference was found among adults and older adults.

## 5. Grain Products

In 2004, Canadians (all energy reporters, all ages combined) consumed, on average, ~5.9 daily servings of total grain products. Whole grains (e.g., whole oats, whole grain brown rice, whole grain pasta) contributed to ~17% of total daily intakes of grain products in both 2004 and 2015. No difference was found in average daily servings of total grain products, whole grains, and non-whole grains among all energy reporters or in analyses which included only plausible energy reporters (all ages combined). In both survey years, mean daily intake of total grain products was highest among children (6–12 years) and adolescents and lowest among young children (2–5 years) and older adults.

The magnitude of the differences in intakes of total grain products over time varied by age group (*p*-value for the interaction <0.05). Age-stratified analyses revealed that children aged 6-12 years (all energy reporters) and both 2–5-year-old and 6–12-year-old children (plausible energy reporters) significantly increased their intakes of total grain products from 2004 to 2015 (+0.7 servings/day). Increases in daily servings of total grain products was largely driven by higher intakes of non-whole grains. Only adolescents (all energy reporters and only plausible reporters) reported significantly higher average daily servings of whole grains in 2015 compared to 2004 (+0.2 servings/day and +0.4 servings/day, respectively). Adults (plausible energy reporters) reported significantly fewer mean daily servings of non-whole grains (−0.2 servings/day) from 2004 to 2015. 

## 6. Milk and Alternatives

In 2004, Canadians (all energy reporters, all ages combined) consumed, on average, ~1.7 servings of milk and alternatives per day. From 2004 to 2015, the amount of milk and alternatives reported significantly decreased by 0.1 servings/day (among all energy reporters and in analyses including only plausible reporters). Canadians (all ages combined) reported consuming significantly more dairy products (i.e., cheese, yogurt) (+0.1 servings/day) while consuming significantly fewer servings of fluid milk from 2004 to 2015 (−0.1 servings/day and −0.2 servings/day, for all energy reporters and for only plausible reporters, respectively). In both survey cycles, intake of fluid milk and other dairy products was highest among children and adolescents and lowest among adults and older adults.

The magnitude of the change varied by age group for milk and alternatives and for fluid milk (*p*-value for the overall interactions both <0.05) in analyses including all energy reporters. Children aged 6–12 years (all energy reporters) reported significantly fewer daily servings of milk and alternatives (−0.1 servings/day) in 2015 compared to 2004, while other age groups reported no difference over time. All age groups reported a statistically significant reduction in daily servings of fluid milk in 2015 compared to 2004.

## 7. Meat and Alternatives

In 2004, Canadians (all energy reporters, all ages combined) consumed, on average, ~2.2 daily servings of meat and alternatives. The largest contributor to meat and alternatives were meat and poultry (~55% and ~50% of total daily intake of meat and alternatives in 2004 and 2015, respectively). In 2004, the second largest contributors to meat and alternatives were legumes, nuts, and seeds combined and processed meats (each contributing to ~14% of meat and alternatives consumed daily). In 2015, the second largest contributor to meat and alternatives were legumes, nuts, and seeds combined (~17% of total daily servings of meat and alternatives consumed). From 2004 to 2015, Canadians (all age groups combined) reported consuming significantly more meat and alternatives (+0.2 servings/day among all energy reporters and among only plausible reporters). Canadians (all age groups combined) reported significant increases in their average daily intake of legumes, nuts, and seeds (+0.1 servings/day) and eggs (+0.1 servings/day among plausible reporters), with no change in other meat and alternatives subgroups. In both survey cycles, mean daily intake of meat and alternatives was highest among adolescents and adults and lowest among children.

The magnitude of the differences in total daily intakes of meat and alternatives varied by age group (*p*-value for the overall interaction <0.001). Adults reported the greatest change in intake of meat and alternatives. Adults reported consuming, on average, significantly more meat and alternatives in 2015 compared to 2004 (+0.3 servings/day and +0.5 servings/day among all energy reporters and among only plausible reporters, respectively). Adults (plausible energy reporters) also reported significantly more meat and poultry (+0.2 servings/day) in 2015. By contrast, older adults reported significantly fewer daily servings of meat and poultry (−0.1 servings/day and −0.3 servings/day among all energy reporters and among only plausible intake reporters, respectively). No significant difference in meat and poultry intake was reported among other age groups.

### 7.1. Mean Daily Intakes from Other Foods and Beverages: 2004 vs. 2015

In 2004, Canadians (all energy reporters, all ages combined) reported consuming, on average, 126 kcal/day from high fat and/or high sugar foods as well as 83 and 22 daily kcal from high- and low-calorie beverages, respectively. From 2004 to 2015, Canadians (all energy reporters, all age groups combined) consumed significantly fewer kcal from high-calorie beverages (for, e.g., non-diet sodas, fruit drinks, sweetened iced tea) (−32 kcal/day), a 39% reduction compared to what was reported a decade earlier. Similar reductions in energy from high-calorie beverages were reported among plausible reporters.

The magnitude of the reduction in high-calorie beverage intake varied by age group (*p*-value for the overall interaction <0.001), but all age groups reported significantly fewer mean daily calories from these beverages from 2004 to 2015. For example, children aged 6-12 years and adolescents (all energy reporters) decreased their average daily calories from these beverages by 58 kcal/day and 73 kcal/day from 2004 to 2015, respectively. Meanwhile, adults and older adults (all energy reporters) reduced their caloric intake from such beverages by only 30 and 12 kcal/day, respectively.

### 7.2. Relative Percent Change in Food and Beverages Intake from 2004 to 2015

**Figure 2:**
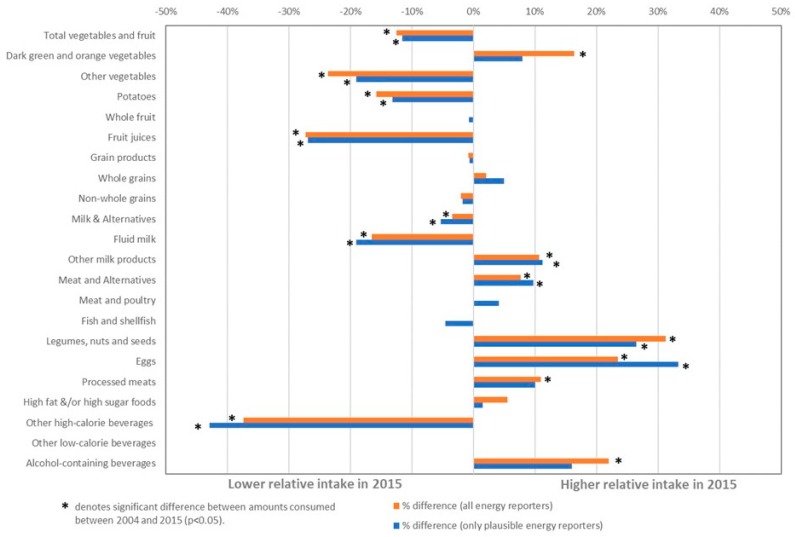
Relative percent (%) change in mean daily intakes of foods among Canadians age 2 years and older ^1^ between 2004 and 2015. ^1^ Average intake from alcohol-containing beverages estimated for respondents aged 13 years and older only.

Average relative percent differences in food group intakes between 2004 and 2015 estimated for all energy reporters and among only plausible energy reporters (all age groups combined) are shown in Figure 2. For 11 out of 22 dietary variables examined, the relative changes in daily intakes were small (i.e., less than a 10% relative difference). However, daily intakes of dark green and orange vegetables, other milk products (i.e., cheese, yogurt), legumes, nuts and seeds, eggs, and alcoholic beverages all increased, on average, by at least 10% or more from 2004 to 2015 (all energy reporters). By contrast, mean daily intakes of total vegetables and fruit, potatoes, other vegetables, fruit juices, fluid milk, and high-calorie beverages all declined by at least 10% over the same time period. For some food subgroups such as fruit juices and high-calorie beverages, these changes were substantial (>25% relative difference), which translated into a ~0.2 fewer daily servings of fruit juices and ~32 fewer daily calories from sugary beverages. Although the relative percent change was large for legumes, nuts and seeds (a 31% increase), and eggs (a 24% increase), the absolute differences over time were small (a difference of ≤0.1 servings/day of meat and alternatives).

## 8. Discussion

This study compared differences in food and beverage intake estimated from nationally representative surveys of Canadians collected in 2004 and 2015. While some aspects of the Canadian diet improved over time (for example, fewer daily kcal from sugar-sweetened beverages), mean daily intake of several types of foods recommended as part of the 2007 CFG and the newly released 2019 Canada’s Dietary Guidelines [7] (e.g., vegetables, whole fruit, whole grains) have either stagnated or worsened over time. These findings are in line with Canadian food balance sheet data which suggest that between 2004 and 2013, the availability of vegetables, milk products, sugars, and sweeteners declined while that of eggs, pulses, and legumes increased [29]. Finally, results found here are also consistent with recent CCHS analyses documenting declines in Canadians’ intake of fluid milk and sugar-sweetened beverages from 2004 to 2015 [11].

We found that Canadians aged 2 years and older reported significantly fewer daily calories consumed in 2015 compared to 2004, and this finding was consistent before and after excluding under- and overreporters. These results are in line with other national-level analyses which have reported changes to energy reporting status and average energy intakes by Canadians from 2004 to 2015 [11]. These findings also echo US research suggesting little change in population-level BMI alongside small declines in population-level energy intakes from 2003–2004 to 2009–2010 [30].

From 2004 to 2015, Canadians reported fewer daily servings of vegetables and fruit, and milk and alternatives and less energy from high-calorie beverages, while reporting higher intakes of meat and alternatives. Excluding under- and overreporters and adjusting for covariates (daily energy intake and other sociodemographic shifts from 2004 to 2015) did not change the direction and statistical significance of these differences. The release of the 2007 CFG took place between the survey years examined here. Some of the differences reported over time were consistent with dietary guidelines issued in the 2007 CFG, including increased daily servings of dark green and orange vegetables, legumes, nuts, and seeds and less energy from high-calorie beverages. The shift towards consuming more plant-based proteins (more legumes, nuts, and seeds) was also in line with recent calls to emphasize more plant-based and environmentally sustainable sources of protein, in Canada [7] and globally [31]. Still, the average amount consumed of many healthful dietary components emphasized in the 2007 CFG (whole fruit, whole grains, fish and shellfish) did not increase over time.

The shift towards consuming fewer servings of fluid milk could be concerning from a population health perspective if fluid milk is not replaced with other food sources rich in calcium and vitamin D [32]. In 2004, national-level analyses suggested that a large proportion of Canadians were not meeting their age and sex-based recommended daily servings for milk and alternatives recommended by the 2007 CFG [4]. Consequently, in 2004, many Canadian adolescents [33], adults [34] and older adults [35] had inadequate intakes of calcium and vitamin D. While our study did not explicitly examine nutrient adequacy (which would have required modelling usual intake distributions), the declines found in population-level mean intakes of milk and alternatives and fluid milk suggests that improvement in the adequacy of these nutrients of concern is unlikely and merits further attention.

Apart from dark green and orange vegetables, estimated intakes of vegetables and fruit generally either stagnated or decreased from 2004 to 2015. In 2004, only 26% of the Canadian population consumed the 2007 CFG minimum number of servings of vegetables and fruit recommended for their respective age–sex group [2], and there were concerns around low intakes of potassium and fibre among both adults [33,34] and children [36]. The population-level decline in vegetables and fruit intake from 2004 to 2015 suggests that more effective efforts are needed to help Canadians move closer towards national dietary recommendations which, as of 2019, encourage having “plenty of vegetables and fruits” [7].

Other international studies examining secular trends in food group intakes demand cautious comparison, as survey methodologies, time frame of data collection, and dietary habits differ across countries. Some international studies drawing from national diet surveys have identified similar shifts in food groups and beverage intakes. In the US, studies drawing from the National Health and Nutrition Examination Surveys suggest that US youth and adults decreased their sugar-sweetened beverages intake by 68 kcal/day and 45 kcal/day from 1999–2000 to 2009–2010, respectively [37]. Similarly, Rehm et al. [38] reported that US adults decreased their mean daily intake of sugar-sweetened beverages by, on average, 0.49 servings/day from 1999 to 2012. Since the beginning of the 21^st^ century, US studies have reported population-level declines in minimally nutritious foods [39,40,41] and *trans* fats [39] but no improvements in other dietary components such as vegetables, dairy products [39,40,41], or sodium [39,40,41]. The differences over time observed in this study were similar in some ways to the US trends: lower amounts of sugar-sweetened beverages, fruit juices, along with little change or decline in some healthier dietary components, such as vegetables and milk and alternatives.

We conducted sensitivity analyses to examine the impact of energy misreporting on differences over time in food group intakes across age groups. In sensitivity analyses that only included plausible energy reporters (all age groups combined), the direction and statistical significance of the temporal difference for most dietary variables (19 out of 22) were consistent with those differences found in analyses which included all energy intake reporters. This suggests that the changes in the proportion of energy misreporting (i.e., greater energy underreporting in 2015 compared to 2004) had little impact overall on food group intake differences reported over time.

These findings suggest some differences in the magnitude of average dietary intake changes over time between children and adults. For example, children aged 2–12 years did not report significantly fewer servings of vegetables and fruit, whereas older age groups did. Moreover, some dietary improvements were observed among younger age groups (e.g., more whole grains among adolescents, more whole fruit among children) but not among adults. These findings are in line with national [41] and international [40,42,43] research suggesting modest improvements in dietary habits of children and adolescents since the early 2000s. For example, in Norway [42] and Scotland [43], data from the Health Behaviour in School-Aged Children study suggested improvement in children’s intakes of fruit, vegetables, and minimally nutritious foods (sweets and sugary beverages) from ~2000 to 2009–2010. In Canada, a recent analysis drawing from the CCHS 2004 and 2015 reported improved school hour and whole school day dietary quality among Canadian children aged 6–17 years [41]. Increased vegetable and fruit intakes and decreased energy from minimally nutritious foods and beverages (observed both during school hours and, to a lesser extent, for the whole school day) accounted for most of the improvement in total diet quality scores over time [41]. This is encouraging, since previous research based on the 2004 CCHS reported that 23–31% of daily calories consumed were derived from other foods and beverages among Canadian children and adolescents [44].

Key strengths of this study include the use of large, nationally representative dietary surveys and the use of 24-h dietary recall data which capture detailed information on the quantity and types of foods consumed. However, there are important limitations that deserve consideration. First, these analyses did not model the distribution in usual intakes. Therefore, it is not possible to report whether the proportion of Canadians who met recommended daily intakes for specific 2007 CFG food groups or the proportion of the population who usually consume no foods from a given subgroup changed over time. Second, this study focused on examining change over time and differences among age groups, but further work is needed to more deeply explore if, how, and why specific dietary practices have changed over time among diverse population groups, including by gender, socioeconomic status or geographical context. Third, since income was measured differently between 2004 and 2015 [45,46], these analyses did not control for income as a potential confounder in these models. It was also not possible to assess national-level changes in food security status between 2004 and 2015 since the CCHS 2015 did not include household-level survey weights [8]. Thus, it is possible that income and/or food security may have acted as potential confounders in the change in dietary variables observed over time. Fourth, there were differences in the execution of the survey (e.g., different sample sizes, response rates, changes to the food booklet used to help estimate portion sizes) and data processing (e.g., changes to the nutrient databases used to analyze the 24-h dietary recalls) between survey cycles, which could have implications when comparing dietary intakes between survey years [8]. For example, a lower response rate (61.6% in 2015 compared to 76% in 2004) increases the potential for non-response bias [8]. There were also changes to the food model booklets regarding the images used to estimate beverage intakes from 2004 to 2015. Line drawings of the glasses, bowls, and plates in 2004 were replaced in 2015 with actual-size photographs that gave a more realistic three-dimensional view of the items, leading potentially to a downward bias when estimating beverage intakes [47,48]. Moreover, differences in energy misreporting (specifically increases in energy underreporting and a decrease in energy overreporting from 2004 to 2015) could alter these findings. However, in sensitivity analyses that only included plausible energy reporters (all age groups combined), the direction and significance of the temporal change for most food/beverage subgroups (19 out of 22) was often consistent with differences found for the full sample. Finally, this analysis did not examine the quality of the food choices over time within each food group and subgroup using the Tier system. For example, we did not examine whether the proportion of Tier 4 foods increased or decreased over time within the food groups examined. Future research is needed to examine how the quality of Canadians’ food choices within food groups have changed over time.

## 9. Conclusions

Small improvements in average food and beverage intakes of Canadians were reported between 2004 to 2015, particularly in terms of a reduction in average daily energy intake from high-calorie beverages and increased intakes of nutritious foods such as dark green and orange vegetables and legumes, nuts, and seeds. However, this study suggests that daily average intake of other nutritious foods has either stagnated (e.g., whole fruit, fish/shellfish, whole grains) or declined (e.g., fluid milk) over time. Future research should focus on understanding the barriers to improving the consumption of “a variety of healthy foods each day” now emphasized in the 2019 Canada’s Dietary Guidelines [7] such as vegetables, whole fruit, whole grain foods, and protein foods such as pulses and nuts, tofu, fish/shellfish, lower fat milk or milk alternatives among Canadians across all life stages.

## Figures and Tables

**Table 1:** Description of the food groups and food subgroups in the 2007 CFG food grouping system.

Food Groups	Food Subgroups	Food Examples with Examples of Serving Sizes	2007 CFG Guidance Statements
Vegetables and fruit	Dark green and orange vegetables	Raw spinach or romaine lettuce (1 cup), sweet potato, carrots (125 mL)	Eat at least one dark green and one orange vegetable each day.
	‘Other’ vegetables	Zucchini, corn, celery, cucumbers (125 mL)	
	Potatoes	Potatoes (baked, mashed, fried) (125 mL)	
	Whole fruit	Bananas, apple, pears, grapes (1 fruit or 125 mL)	Have vegetables and fruit more often than juice.
	Fruit juice	100% orange juice (125 mL)	
Grain products	Non-whole grains	White bread (1 slice or 35 g), white rice (125 mL), pita or tortilla (35 g), corn flakes (35 g)	
	Whole grains	Whole oats or oatmeal (175 mL), whole grain brown or wild rice or pasta (125 mL)	Make at least half of your grain products whole grain each day.
Milk and alternatives	Fluid milk and fortified soy-based beverages	Cow’s milk, fortified soy milk, flavored milk (250 mL)	Drink skim, 1%, or 2% milk each day. Have 500 mL (2 cups) of milk every day for adequate vitamin D.
	Other milk products	Plain or flavored yogurt (175 g), cheese (50 g)	
Meat and alternatives	Beef, pork, veal, lamb, poultry, game and organ meats	Roasted chicken, turkey, ground beef (75 g)	
	Fish and shellfish	Salmon, shrimp	Eat at least two food guide servings of fish each week.
	Legumes, nuts and seeds and soy	Peanut butter (30 mL), cooked legumes (175 mL), shelled nuts and seeds (60 mL), tofu (150 g)	Have meat alternatives such as beans, lentils and tofu often.
	Eggs	Scrambled eggs (2 eggs)	
	Processed meats	Smoked turkey, salami (75 g)	
Other foods ^1^	High fat and/or high sugar foods	Candies, chocolate, syrups, sauces, fruit jams	Limit foods and beverages high in calories, fat, sugar or salt (sodium) such as cakes and pastries, chocolate and candies, cookies and granola bars, doughnuts and muffins, ice cream and frozen desserts, French fries, potato chips, nachos and other salty snacks, alcohol, fruit flavored drinks, soft drinks, sports and energy drinks, and sweetened hot or cold drinks.
Other beverages ^1^	High calorie other beverages (>40 kcal/100 g)	Sodas such as Coca Cola©, Sprite©	
	Low calorie other beverages (≤40 kcal/100 g)	Tea and coffee (unsweetened or lightly sweetened), vitamin water, almond milk	
	Alcohol-containing beverages	Wine, beer	

CFG, Canada’s Food Guide; CNF, Canadian Nutrient File. ^1^ No standard servings existed for other foods and other beverages (foods that were not part of the four core four food groups).

**Table 2:** Energy reporting characteristics among Canadians aged 2 years and older in 2004 and in 2015 (*n* = 20,738 in 2004 and *n* = 13,611 in 2015).

	Survey Cycle			
	2004		2015	
	Mean	SE	Mean	SE
Energy reporting category				
Underreporter (%)	22.3	0.6	30.1 *	0.8
Plausible reporter (%)	60.6	0.7	60.6	0.8
Overreporter (%)	17.1	0.6	9.3 *	0.4

SE, Standard error. * Significant differences between 2004 and 2015 (*p*-value ≤ 0.05). Children and adults with measured weight and height were classified as either under-, plausible or over-energy reporters based on the ratio of reported vs. total energy expenditure (TEE). TEE was based on equations that account for height, weight, age, sex, physical activity levels, and a person’s weight status (normal weight vs. overweight/obese) [22]. Physical activity levels were assumed to be low active for children aged 13 and younger and sedentary for respondents aged 14 and older.

**Table 3:** Sociodemographic and lifestyle characteristics among Canadians aged 2 years and older in 2004 and in 2015 (*n* = 33,514 in 2004 and *n =* 19,408 in 2015).

	Survey Cycle			
	2004		2015	
	Mean	SE	Mean	SE
Mean age (years)	38.6	0.1	41.4 *	0.1
Sex (% male)	50.6	0.1	51.1	0.2
Location of residence (% urban)	82.2	0.5	82.4	0.7
Ethnicity (% White/Caucasian) ^1^	82.6	0.5	76.1 *	0.7
Immigrant status (% immigrant) ^2^	19.8	0.5	23.6 *	0.8
Education (% university graduate) ^3^	34.1	0.6	45.7 *	0.9
Body Mass Index (BMI), adults ≥ 18 years (kg/m^2^) ^4^	27.2	0.1	27.6 *	0.1
Overweight/obese, adults ≥ 18 years (% yes) ^4^	60.8	0.9	63.3	1.1
Overweight/obese, children 2–17 years (% yes) ^5^	26.3	0.8	24.1	1.2
Smoking status (% yes) ^6^	23.7	0.6	17.6 *	0.7
Supplement use in past 30 days (% yes) ^7^	40.1	0.5	45.1 *	0.7

SE, Standard error. * Significant differences between 2004 and 2015 (*p*-value ≤ 0.05). ^1^ Missing data for 949 respondents (1.8% of all respondents in 2004 and 2015). ^2^ Missing data for 62 respondents (0.1% of all respondents in 2004 and 2015). ^3^ Missing data for 599 respondents (1.1% of all respondents in 2004 and 2015). ^4^ Applicable to adults with measured weight and height (*n* = 12,093 in 2004 and *n* = 9139 in 2015). ^5^ Applicable to children with measured weights and heights (*n* = 8645 in 2004 and *n* = 4472 in 2015). ^6^ Applicable to respondents aged 12 years and older (*n* = 26,295 in 2004 and *n* = 15,961 in 2015). ^7^ Missing data for 15 respondents (0.04% of all respondents in 2004 and 2015).

**Table 4:** Differences in average daily energy intake (kcal/day) between 2004 and 2015 among Canadians ≥2 y (all energy reporters and only plausible energy reporters).

	All Energy Reporters						Only Plausible Energy Reporters ^1^					
	Survey Cycle						Survey Cycle					
	2004		2015		Difference		2004		2015		Difference	
	*n* = 33,314		*n* = 19,408				*n* = 12,796		*n* = 8227			
	Mean	SE	Mean	SE	Mean	95% CI	Mean	SE	Mean	SE	Mean	95% CI
**Daily energy intake, kcal**												
Young children, 2–5 y	1681	19	1430	22	−250 *	−306, −195	1496	14	1412	20	−85 *	−132, −37
Children, 6–12 y	2106	20	1849	21	−256 *	−311, −201	1977	15	1870	17	−106 *	−152, −59
Adolescents, 13–17 y	2452	25	2115	37	−337 *	−425, −248	2301	22	2170	25	−131 *	−197, −66
Adults, 18–54 y	2219	18	1977	20	−241 *	−294, −189	2263	18	2194	23	−69 *	−125, −14
Older adults, ≥55 y	1832	15	1752	18	−80 *	−126, −34	1950	15	1940	18	−10	−56, 38
All ages	2108	11	1880	12	−228 *	−260, −195	2123	10	2049	14	−74 *	−107, −41

SE, Standard error. CI, Confidence interval. Y, years. The data are weighted for the Canadian population, but unweighted sample sizes are shown. * Significant difference from 2004 to 2015 (*p*-value ≤ 0.05). Differences in average daily energy intake were estimated from simple linear models. ^1^ Children and adults with measured weight and height were classified as either under-, plausible or over-energy reporters based on the ratio of reported vs. total energy expenditure (TEE). TEE was based on equations that account for height, weight, age, sex, physical activity levels, and person’s weight status (normal weight vs. overweight/obese) [22]. Physical activity levels were assumed to be low active for children aged 13 and younger and sedentary for respondents aged 14 and older.

**Table 5:** Difference in covariate-adjusted mean daily amounts of foods and beverages reported between 2004 and 2015 among Canadians ≥2 y (all energy reporters and only plausible energy reporters).

	All Energy Reporters						Only Plausible Energy Reporters ^1^					
	Survey Cycle						Survey Cycle					
	2004		2015		Difference		2004		2015		Difference	
	*n* = 32,890		*n* = 18,447				*n* = 12,602		*n* = 7833			
	Mean	SE	Mean	SE	Mean	95% CI	Mean	SE	Mean	SE	Mean	95% CI
**Total vegetables and fruit, servings**												
Young children, 2–5 y	4.2	0.1	4.2	0.1	0.0	−0.3, 0.3	4.3	0.1	3.9	0.1	−0.3	−0.7, 0.1
Children, 6–12 y	4.5	0.1	4.3	0.1	−0.1	−0.4, 0.1	4.4	0.1	4.5	0.1	0.1	−0.3, 0.4
Adolescents, 13–17 y	4.9	0.1	4.5	0.1	−0.4 *	−0.7, −0.0	4.9	0.1	4.5	0.2	−0.5	−0.9, 0.0
Adults, 18–54 y	5.4	0.1	4.6	0.1	−0.8 *	−1.0, −0.6	5.6	0.1	4.7	0.1	−0.9 *	−1.2, −0.6
Older adults, ≥55 y	5.5	0.1	4.7	0.1	−0.8 *	−1.0, −0.6	5.7	0.1	5.1	0.1	−0.6 *	−0.9, −0.3
All ages	5.2	0.0	4.6	0.0	−0.7 *^,†^	−0.8, −0.5	5.4	0.1	4.7	0.1	−0.6 *^,†^	−0.8, −0.4
**Dark green and orange vegetables, servings**												
Young children, 2–5 y	0.3	0.0	0.4	0.0	0.1	0.0, 0.2	0.3	0.0	0.3	0.0	0.0	−0.0, 0.1
Children, 6–12 y	0.4	0.0	0.4	0.0	0.0	−0.0, 0.1	0.4	0.0	0.4	0.0	0.1 *	0.0, 0.1
Adolescents, 13–17 y	0.4	0.0	0.5	0.0	0.1 *	0.0, 0.2	0.4	0.0	0.5	0.0	0.1	−0.0, 0.2
Adults, 18–54 y	0.6	0.0	0.8	0.0	0.1 *	0.1, 0.2	0.7	0.0	0.7	0.0	0.1	−0.0, 0.2
Older adults, ≥55 y	0.7	0.0	0.7	0.0	0.0	−0.1, 0.1	0.8	0.0	0.8	0.0	0.0	−0.1, 0.1
All ages	0.6	0.0	0.7	0.0	0.1 *	0.0, 0.1	0.6	0.0	0.7	0.0	0.1	−0.0, 0.1
**Other vegetables, servings**												
Young children, 2–5 y	0.7	0.0	0.7	0.0	0.0	−0.1, 0.1	0.7	0.1	0.7	0.1	0.0	−0.2, 0.2
Children, 6–12 y	1.0	0.0	0.9	0.0	0.0	−0.1, 0.1	0.9	0.0	1.0	0.1	0.1 *	0.0, 0.3
Adolescents, 13–17 y	1.3	0.0	1.0	0.0	−0.2 *	−0.4, −0.1	1.2	0.1	1.0	0.1	−0.2 *	−0.4, −0.1
Adults, 18–54 y	1.8	0.0	1.4	0.0	−0.5 *	−0.6, −0.4	1.9	0.1	1.4	0.1	−0.5 *	−0.6, −0.3
Older adults, ≥55 y	1.8	0.0	1.3	0.0	−0.5 *	−0.6, −0.3	1.8	0.1	1.5	0.1	−0.3 *	−0.5, −0.2
All ages	1.7	0.0	1.3	0.0	−0.4 *^,†^	−0.5, −0.3	1.6	0.0	1.3	0.0	−0.3 *^,†^	−0.4, −0.2
**Potatoes, servings**												
Young children, 2–5 y	0.4	0.0	0.3	0.0	−0.1 *	−0.2, −0.0	0.4	0.0	0.3	0.0	−0.1	−0.2, 0.0
Children, 6–12 y	0.7	0.0	0.5	0.0	−0.2 *	−0.2, −0.1	0.7	0.0	0.5	0.0	−0.2 *	−0.3, −0.1
Adolescents, 13–17 y	0.9	0.0	0.7	0.1	−0.2 *	−0.3, −0.1	0.8	0.0	0.7	0.1	−0.2 *	−0.3, −0.0
Adults, 18–54 y	0.8	0.0	0.6	0.0	−0.2 *	−0.2, −0.1	0.8	0.0	0.6	0.0	−0.1 *	−0.2, −0.0
Older adults, ≥55 y	0.8	0.0	0.8	0.0	0.0	−0.1, 0.1	0.8	0.0	0.8	0.0	0.0	−0.1, 0.2
All ages	0.8	0.0	0.6	0.0	−0.1 *	−0.2, −0.1	0.8	0.0	0.7	0.0	−0.1 *	−0.2, −0.0
**Whole fruit, servings**												
Young children, 2–5 y	1.3	0.1	1.8	0.1	0.4 *	0.2, 0.6	1.5	0.1	1.6	0.1	0.2	−0.1, 0.5
Children, 6–12 y	1.3	0.1	1.5	0.1	0.2 *	0.0, 0.3	1.4	0.1	1.5	0.1	0.2	−0.1, 0.4
Adolescents, 13–17 y	1.0	0.4	1.3	0.1	0.2 *	0.0, 0.4	1.0	0.1	1.3	0.1	0.2 *	0.0, 0.5
Adults, 18–54 y	1.3	0.0	1.2	0.0	−0.1	−0.2, 0.1	1.4	0.1	1.3	0.1	−0.1	−0.3, 0.1
Older adults, ≥55 y	1.5	0.0	1.4	0.0	−0.1	−0.2, 0.0	1.5	0.1	1.5	0.1	−0.1	−0.2, 0.1
All ages	1.3	0.0	1.3	0.0	0.0 ^†^	−0.1, 0.1	1.4	0.1	1.4	0.0	0.0 ^†^	−0.1, 0.1
**Fruit juices, servings**												
Young children, 2–5 y	1.4	0.1	1.0	0.1	−0.4 *	−0.6, −0.2	1.4	0.1	0.9	0.1	−0.4 *	−0.7, −0.2
Children, 6–12 y	1.2	0.0	1.0	0.0	−0.1 *	−0.3, −0.0	1.2	0.1	1.0	0.1	−0.1	−0.3, 0.0
Adolescents, 13–17 y	1.3	0.1	1.0	0.1	−0.3 *	−0.5, −0.1	1.5	0.1	1.1	0.1	−0.4 *	−0.7, −0.1
Adults, 18–54 y	0.8	0.0	0.6	0.0	−0.2 *	−0.3, −0.2	0.9	0.1	0.6	0.1	−0.2 *	−0.4, −0.1
Older adults, ≥55 y	0.7	0.0	0.4	0.0	−0.2 *	−0.3, −0.1	0.7	0.0	0.5	0.0	−0.3 *	−0.4, −0.2
All ages	0.9	0.0	0.6	0.0	−0.2 *^,†^	−0.3, −0.2	0.9	0.0	0.7	0.0	−0.2 *	−0.3, −0.2
**Total grain products, servings**												
Young children, 2–5 y	4.5	0.1	4.6	0.1	−0.2	−0.1, 0.4	4.3	0.1	4.6	0.1	0.3 *	0.0, 0.7
Children, 6–12 y	6.4	0.1	7.1	0.1	0.7 *	0.4, 0.9	6.2	0.1	6.9	0.1	0.7 *	0.5, 1.0
Adolescents, 13–17 y	7.2	0.1	7.2	0.1	0.1	−0.2, 0.3	7.1	0.1	7.3	0.1	0.1	−0.3, 0.5
Adults, 18–54 y	6.1	0.1	5.9	0.1	−0.2	−0.4, 0.0	6.6	0.1	6.3	0.1	−0.3	−0.6, 0.1
Older adults, ≥55 y	5.2	0.1	5.1	0.1	−0.1	−0.3, 0.1	5.7	0.1	5.7	0.1	−0.1	−0.3, 0.2
All ages	5.9	0.0	5.8	0.0	0.0 ^†^	−0.2, 0.1	6.2	0.1	6.2	0.1	0.0 ^†^	−0.2, 0.2
**Whole grains, servings**												
Young children, 2–5 y	0.7	0.0	0.8	0.1	0.0	−0.1, 0.2	0.6	0.0	0.8	0.1	0.2	−0.0, 0.3
Children, 6–12 y	0.9	0.0	1.0	0.0	0.1	−0.0, 0.2	1.0	0.1	1.1	0.1	0.1	−0.1, 0.3
Adolescents, 13–17 y	0.9	0.0	1.1	0.1	0.2 *	0.0, 0.4	0.9	0.1	1.3	1.1	0.4 *	0.1, 0.6
Adults, 18–54 y	1.0	0.0	1.0	0.0	0.0	−0.2, 0.1	1.0	0.1	1.0	0.1	0.0	−0.1, 0.2
Older adults, ≥55 y	1.1	0.0	1.0	0.0	-0.1	−0.2, 0.0	1.2	0.1	1.1	0.1	−0.1	−0.2, 0.1
All ages	1.0	0.0	1.0	0.0	0.0 ^†^	−0.0, 0.1	1.0	0.0	1.1	0.0	0.1	0.0, 0.2
**Non-whole grains, servings**												
Young children, 2–5 y	3.7	0.1	3.8	0.1	0.1	−0.1, 0.3	3.6	0.1	3.8	0.1	0.2	−0.2, 0.5
Children, 6–12 y	5.5	0.1	6.0	0.1	0.5 *	0.3, 0.8	5.2	0.1	5.8	0.1	0.6 *	0.3, 0.9
Adolescents, 13–17 y	6.2	0.1	6.1	0.1	−0.1	−0.4, 0.2	6.2	0.1	6.0	0.1	−0.2	−0.4, 0.2
Adults, 18–54 y	6.2	0.1	6.0	0.2	−0.2	−0.6, 0.2	5.2	0.1	5.0	0.1	−0.2 *	−0.4, −0.0
Older adults, ≥55 y	4.1	0.1	4.1	0.1	0.0	−0.2, 0.1	4.5	0.1	4.5	0.1	0.0	−0.3, 0.3
All ages	4.9	0.0	4.8	0.0	−0.1	−0.2, 0.0	5.2	0.1	5.1	0.1	−0.1 ^†^	−0.3, 0.1
**Milk & alternatives, servings**												
Young children, 2–5 y	2.4	0.0	2.5	0.1	0.1	−0.1, 0.2	2.2	0.1	2.3	0.1	0.1	−0.2, 0.3
Children, 6–12 y	2.5	0.0	2.3	0.0	−0.1 *	−0.3, −0.0	2.4	0.1	2.3	0.1	-0.1	−0.3, 0.1
Adolescents, 13–17 y	2.4	0.0	2.4	0.1	0.0	−0.1, 0.2	2.4	0.1	2.3	0.1	-0.1	−0.3, 0.2
Adults, 18–54 y	1.7	0.0	1.6	0.0	-0.1	−0.2, 0.0	1.8	0.0	1.7	0.1	−0.1	−0.3, 0.0
Older adults, ≥55 y	1.4	0.0	1.4	0.0	0.0	−0.1, 0.1	1.6	0.0	1.5	0.0	0.0	−0.1, 0.1
All ages	1.7	0.0	1.7	−0.1	−0.1 *^,†^	−0.1, −0.0	1.9	0.0	1.8	0.0	−0.1 *	−0.2, -0.0
**Fluid milk, servings**												
Young children, 2–5 y	1.6	0.0	1.4	0.1	−0.2 *	−0.3, −0.0	1.4	0.1	1.2	0.1	−0.2 *	−0.4, −0.0
Children, 6–12 y	1.4	0.0	1.1	0.0	−0.3 *	−0.4, −0.2	1.4	0.0	1.1	0.0	−0.3 *	−0.4, −0.1
Adolescents, 13–17 y	1.4	0.0	1.2	0.0	−0.2 *	−0.3, −0.1	1.4	0.1	1.1	0.1	−0.2 *	−0.4, −0.1
Adults, 18–54 y	0.8	0.0	0.7	0.0	−0.1 *	−0.2, −0.1	0.9	0.0	0.7	0.0	−0.2 *	−0.3, −0.1
Older adults, ≥55 y	0.7	0.0	0.6	0.0	−0.2 *	−0.2, −0.1	0.8	0.0	0.7	0.0	−0.2 *	−0.3, −0.1
All ages	0.9	0.0	0.8	0.0	−0.1 *^,†^	−0.2, −0.1	1.0	0.0	0.8	0.0	−0.2 *	−0.2, −0.1
**Other milk products (yogurt, cheese), servings**												
Young children, 2–5 y	0.9	0.0	1.1	0.1	0.2 *	0.1, 0.3	0.8	0.0	1.0	0.1	0.3 *	0.1, 0.4
Children, 6–12 y	1.0	0.0	1.2	0.0	0.1 *	0.1, 0.2	1.0	0.0	1.1	0.0	0.2 *	0.0, 0.3
Adolescents, 13–17 y	1.0	0.0	1.2	0.1	0.2 *	0.1, 0.3	1.0	0.0	1.1	0.1	0.2	−0.1, 0.3
Adults, 18–54 y	0.9	0.0	0.9	0.0	0.0	−0.0, 0.1	0.9	0.0	1.0	0.0	0.0	−0.1, 0.2
Older adults, ≥55 y	0.7	0.0	0.8	0.0	0.1 *	0.1, 0.2	0.8	0.0	0.9	0.0	0.1 *	0.0, 0.2
All ages	0.8	0.0	0.9	0.0	0.1 *	0.0, 0.1	0.9	0.0	1.0	0.0	0.1 *	0.0, 0.2
**Meat and alternatives, servings**												
Young children, 2–5 y	1.1	0.0	1.3	0.1	0.2 *	0.1, 0.4	1.0	0.0	1.1	0.1	0.1	−0.0, 0.3
Children, 6–12 y	1.6	0.0	1.6	0.0	0.0	−0.1, 0.1	1.6	0.0	1.6	0.1	0.0	−0.1, 0.1
Adolescents, 13–17 y	2.1	0.0	2.3	0.1	0.2 *	0.0, 0.3	2.0	0.1	2.2	0.1	0.2	−0.0, 0.4
Adults, 18–54 y	2.5	0.0	2.7	0.0	0.3 *	0.2, 0.4	2.5	0.1	2.9	0.1	0.5 *	0.2, 0.7
Older adults, ≥55 y	2.2	0.0	2.2	0.0	0.0	−0.1, 0.1	2.4	0.0	2.3	0.1	−0.2 *	−0.3, −0.0
All ages	2.2	0.0	2.4	0.0	0.2 *^,†^	0.1, 0.2	2.3	0.0	2.5	0.0	0.2 *^,†^	0.1, 0.3
**Meat and poultry, servings**												
Young children, 2–5 y	0.5	0.0	0.6	0.0	0.0	−0.0, 0.1	0.5	0.0	0.5	0.1	0.0	−0.1, 0.1
Children, 6–12 y	0.9	0.0	0.8	0.0	−0.1	−0.1, 0.0	0.8	0.0	0.8	0.0	−0.1	−0.2, 0.0
Adolescents, 13–17 y	1.3	0.0	1.3	0.1	0.0	−0.1, 0.1	1.2	0.1	1.2	0.1	0.0	−0.2, 0.1
Adults, 18–54 y	1.4	0.0	1.4	0.0	0.0	−0.1, 0.2	1.3	0.1	1.6	0.1	0.2 *	0.0, 0.5
Older adults, ≥55 y	1.1	0.0	1.0	0.0	−0.1 *	−0.2, −0.0	1.3	0.0	1.0	0.0	−0.3 *	−0.4, −0.1
All ages	1.2	0.0	1.2	0.0	0.0 ^†^	−0.1, 0.1	1.2	0.0	1.3	0.0	0.1 ^†^	−0.1, 0.2
**Fish and shellfish, servings**												
Young children, 2–5 y	0.1	0.0	0.1	0.0	0.1 *	0.0, 0.1	0.1	0.0	0.1	0.0	0.1	0.0, 0.1
Children, 6–12 y	0.1	0.0	0.1	0.0	0.0	0.0, 0.0	0.1	0.0	0.1	0.0	0.0	−0.1, 0.0
Adolescents, 13–17 y	0.1	0.0	0.1	0.0	0.0	0.0, 0.1	0.1	0.0	0.1	0.0	0.0	−0.0, 0.1
Adults, 18–54 y	0.2	0.0	0.2	0.0	0.0	−0.0, 0.0	0.2	0.0	0.2	0.0	0.0	−0.1, 0.0
Older adults, ≥55 y	0.3	0.0	0.3	0.0	0.0	−0.1, 0.0	0.3	0.0	0.3	0.0	0.0	−0.1, 0.0
All ages	0.2	0.0	0.2	0.0	0.0 ^†^	−0.0, 0.0	0.2	0.0	0.2	0.0	0.0	−0.0, 0.0
**Legumes, nuts and seeds, servings**												
Young children, 2–5 y	0.1	0.0	0.2	0.0	0.1	−0.0, 0.1	0.1	0.0	0.1	0.0	0.0	−0.0, 0.1
Children, 6–12 y	0.2	0.0	0.2	0.0	0.0	−0.1, 0.0	0.2	0.0	0.2	0.0	0.0	0.0, 0.1
Adolescents, 13–17 y	0.2	0.0	0.3	0.0	0.1 *	0.0, 0.2	0.2	0.0	0.3	0.1	0.1	−0.0, 0.3
Adults, 18–54 y	0.4	0.0	0.5	0.0	0.1 *	0.1, 0.2	0.4	0.0	0.5	0.0	0.1 *	0.0, 0.2
Older adults, ≥55 y	0.3	0.0	0.4	0.0	0.1	−0.0, 0.1	0.4	0.0	0.4	0.0	0.1	−0.0, 0.2
All ages	0.3	0.0	0.4	0.0	0.1 *	0.1, 0.1	0.3	0.1	0.4	0.0	0.1 *	0.0, 0.1
**Eggs, servings**												
Young children, 2–5 y	0.1	0.0	0.1	0.0	0.0	−0.0, 0.0	0.1	0.0	0.0	0.0	0.0	−0.0, 0.0
Children, 6–12 y	0.1	0.0	0.1	0.0	0.0	−0.0, 0.0	0.1	0.0	0.1	0.0	0.0	−0.0, 0.1
Adolescents, 13–17 y	0.1	0.0	0.2	0.0	0.1 *	0.0, 0.1	0.1	0.0	0.2	0.0	0.1 *	0.0, 0.1
Adults, 18–54 y	0.2	0.0	0.3	0.0	0.1 *	0.0, 0.1	0.2	0.0	0.3	0.0	0.1 *	0.0, 0.1
Older adults, ≥55 y	0.2	0.0	0.2	0.0	0.0 *	0.0, 0.1	0.2	0.0	0.2	0.0	0.0	−0.0, 0.1
All ages	0.2	0.0	0.2	0.0	0.0 *	0.0, 0.1	0.2	0.0	0.2	0.0	0.1 *	0.0, 0.1
**Processed meats, servings**												
Young children, 2–5 y	0.2	0.0	0.3	0.0	0.1	−0.0, 0.2	0.2	0.0	0.3	0.0	0.0	−0.1, 0.1
Children, 6–12 y	0.3	0.0	0.3	0.0	0.0	−0.0, 0.0	0.3	0.0	0.4	0.0	0.0	−0.0, 0.1
Adolescents, 13–17 y	0.4	0.0	0.4	0.0	0.0	−0.0, 0.1	0.4	0.0	0.4	0.0	0.0	−0.1, 0.1
Adults, 18–54 y	0.3	0.0	0.3	0.0	0.0	−0.0, 0.1	0.3	0.0	0.3	0.0	0.0	−0.0, 0.1
Older adults, ≥55 y	0.2	0.0	0.3	0.0	0.0	−0.0, 0.1	0.3	0.0	0.3	0.0	0.0	−0.0, 0.1
All ages	0.3	0.0	0.3	0.0	0.0 *	0.0, 0.1	0.3	0.0	0.3	0.0	0.0	−0.0, 0.1
**High fat &/or high sugar foods, kcal**												
Young children, 2–5 y	89	5	88	5	−2	−15, 12	82	6	93	8	11	−9, 32
Children, 6–12 y	153	4	148	6	5	−9, 19	145	6	131	7	−12	−29, 6
Adolescents, 13–17 y	166	7	178	10	12	−11, 35	145	7	157	10	12	−14, 37
Adults, 18–54 y	132	3	138	5	6	−6, 19	143	6	140	6	−3	−19, 13
Older adults, ≥55 y	104	3	115	4	12 *	3, 21	112	4	125	6	13	−2, 28
All ages	126	2	133	3	7 ^†^	0, 15	132	3	134	4	2	−8, 11
**High-calorie beverages (>40 kcal/100g), kcal**												
Young children, 2–5 y	66	3	17	2	−49 *	−57, −42	57	4	17	3	−41 *	−51, −31
Children, 6–12 y	108	3	51	3	−58 *	−65, −50	109	4	46	3	−63 *	−73, −52
Adolescents, 13–17 y	169	5	96	6	−73 *	−88, −58	159	6	90	7	−69 *	−88, −50
Adults, 18–54 y	91	2	62	3	−30 *	−37, −21	103	5	64	5	−38 *	−51, −25
Older adults, ≥55 y	40	2	28	2	−12 *	−17, −6	42	3	26	2	−16 *	−23, −9
All ages	83	2	52	2	−32 *^,†^	−36, −27	91	2	52	2	−39 *^,†^	−46, −32
**Low-calorie beverages (≤40 kcal/100 g), kcal**												
Young children, 2–5 y	9	1	8	2	−1	−5, 2	9	2	11	3	2	−4, 8
Children, 6–12 y	15	1	17	1	2	−2, 5	15	1	14	1	−1	−5, 3
Adolescents, 13–17 y	25	2	33	3	8 *	2, 15	25	3	36	4	11 *	1, 20
Adults, 18–54 y	25	1	25	2	−1	−6, 6	26	2	25	4	−1	−12, 10
Older adults, ≥55 y	19	1	18	1	−1	−4, 2	20	2	21	2	1	−5, 6
All ages	22	1	22	1	0	−3, 3	23	1	23	2	0	−6, 6
**Alcohol-containing beverages, kcal**												
Adolescents, 13–17 y	5	1	7	2	2	−4, 8	4	1	3	1	−1	−5, 2
Adults, 18–54 y	88	4	101	6	13	−1, 27	92	6	105	8	12	−8, 32
Older adults, ≥55 y	63	3	89	5	26 *	15, 37	73	6	88	8	15	−4, 34
All ages	74	2	90	4	16 *	7, 25	79	4	91	6	13	−1, 26

CI, Confidence intervals. SE, Standard error. Y, years. Data were weighted for the Canadian population, but unweighted sample sizes are shown. ^1^ Children and adults with measured weight and height were classified as either under-, plausible or over-energy reporters based on the ratio of reported vs. total energy expenditure (TEE). TEE was based on equations that account for height, weight, age, sex, physical activity levels, and person’s weight status (normal weight vs. overweight/obese) [22]. Physical activity levels were assumed to be low active for children aged 13 and younger and sedentary for respondents aged 14 and older. * Significant difference between 2004 and 2015 was tested using multivariable linear regression models adjusted for daily energy intake, age in years, ethnicity, immigrant status, household-level education, smoking status, and supplement use. Sample sizes differ slightly from the unadjusted models due to missing data for ethnicity, immigrant status, household-level education, and supplement use. Due to large numbers of missing data for the smoking variable (“not applicable” for respondents under 12 years, “refused”, “don’t know” or “not stated”), a dummy variable was created to avoid dropping these respondents in covariate-adjusted linear models. ^†^ *p*-value from the Wald test testing the joint significance of adding the age group and survey year interaction product terms is significant (*p*-value < 0.05).
