# Correction: Tugault-Lafleur C.N. and Black J.L. “Differences in the Quantity and Types of Foods and Beverages Consumed by Canadians between 2004 and 2015” *Nutrients* 2019, *11*, 526

**DOI:** 10.3390/nu11092160

**Published:** 2019-09-09

**Authors:** Claire N. Tugault-Lafleur, Jennifer L. Black

**Affiliations:** Food, Nutrition and Health Program, 2205 East Mall, The University of British Columbia, Vancouver, BC V6T 1Z4, Canada

We would like to submit the following corrections to our recently published paper [1] because a coding error was identified in Statistic Canada’s assignment of vegetable subgroup categories for 21 vegetables in the 2004 Canadian Community Health Survey (CCHS) data files. Specifically, vegetables classified as dark green and orange using the 2007 Canada’s Food Guide classifications (e.g., asparagus, snow peas, peas, and carrots) were coded as “non-dark green and orange” in the 2004 CCHS dataset, but correctly coded as “dark green and orange” in the 2015 CCHS dataset. Since this paper assessed changes in intake in dark green and orange vegetables between 2004 and 2015 (and, therefore, requires consistent coding across years to generate valid estimates of difference), we recalculated the difference over time after manually recoding the 2004 items to match the definitions and coding used in the 2015 data files.

In the original paper, we reported that Canadians (2 years and older) had reportedly **increased**, on average, their amount of dark green and orange vegetables consumed by 0.1 servings daily from 2004 to 2015. Instead, the corrected results suggest that Canadians **decreased** their average daily servings of dark green and orange vegetables by 0.1 servings daily. The revised coding also impacted the difference estimated over time in the intake of non-dark green and orange vegetables (i.e., “other” vegetables). In the original paper, we reported that Canadians (ages 2 years and older) decreased their daily intake of other vegetables by 0.4 servings daily. The corrected results suggest that Canadians decreased average intake of other vegetables by only 0.2 servings per day. No other coding errors were found among other food groups or subgroups examined in this paper. In the following text, we provide updated results and text using the more appropriate coding for dark green and orange vegetables.

**Abstract:** The sentence that stated “Analyses of food subgroups revealed that Canadians reported consuming more daily servings of dark green and orange vegetables […] in 2015 compared to 2004” should state that “Analyses of food subgroups revealed that Canadians reported consuming more daily servings of dairy products, legumes, nuts and seeds, and eggs but fewer servings of potatoes, vegetables (**dark green and orange** and “other” vegetables), fruit juices, fluid milk, and sugar-sweetened beverages in 2015 compared to 2004.”

## Results:

### Vegetables and Fruit

On page 8 of the original paper, the sentence: “In 2004, the largest contributor to total vegetables and fruit were “other” vegetables (i.e., non-dark green and orange vegetables such as cucumbers, tomatoes, celery, corn), which represented ~33% of total daily servings of vegetables and fruit” should state: “In 2004, the largest contributor to total vegetables and fruit were “other” vegetables […], which represented ~**29**% of total daily servings of vegetables and fruit”.

The revised values for total vegetable and fruit and vegetable and fruit subgroups are shown below in Table 1. The corrected mean amounts of dark green and orange vegetables and “other” vegetables for 2004 and 2015, as well as the corrected mean difference over time are highlighted in **bold**. Revisions did not impact the difference in total vegetables and fruit servings over time. There were no other changes in other food groups or subgroups (grain products, milk and alternatives, and meat and alternatives).

In the original paper on page 8, we stated that the magnitude of the reduction in some categories of vegetables and fruit varied by age groups (e.g., “other” vegetables, whole fruit, and fruit juices (among all energy reporters)). After correcting for the coding errors, the magnitude of difference in the daily amount of dark green and orange vegetables also varied by age group. Specifically, children aged 6–12 years (all energy reporters only) and adults and older adults (both for the full sample and among plausible respondents) reported significantly fewer average daily servings of dark green and orange vegetables in 2015 compared to 2004, whereas young children and adolescents reported no differences over time.

### Relative Percent Change in Food and Beverages Intake from 2004 to 2015

The coding errors affected the relative percent changes estimated for dark green and orange vegetables and other vegetables reported by Canadians from 2004 to 2015, discussed on page 18 in the original paper. The revised Figure 1 is shown below. Revised findings suggest that Canadians aged 2 years and older reported consuming at least 15% fewer dark green and orange vegetables from 2004 to 2015. Over the same time period, Canadians reported consuming 8% and 13% fewer “other” vegetables, among plausible reporters and among all energy reporters, respectively.

## Discussion

In the third paragraph, the Discussion should read as follows: “Still, the average consumption of many healthful dietary components emphasized in the 2007 CFG (dark green and orange vegetables, whole fruit, whole grains, fish, and shellfish) did not increase over time.”

The first line of the fifth paragraph should now read as follows: “Apart from whole fruit, which did not change over time, the estimated average daily intakes of **dark green and orange vegetables**, “other” vegetables, potatoes, and fruit juice all decreased from 2004 to 2015.”

Finally, the last paragraph of the Discussion should read as follows: “However, in sensitivity analyses that only included plausible energy reporters (all age groups combined), the direction and significance of the temporal change for most food/beverage subgroups (**20** out of 22) were consistent with differences found for the full sample.”

## Conclusions

This first two sentences of the Conclusion paragraph should now read: “Small improvements in average food and beverage intakes of Canadians were reported between 2004 to 2015, particularly in terms of a reduction in average daily energy intake from high-calorie beverages and increased intakes of nutritious foods such as legumes, nuts, and seeds. However, this study suggests that the daily average intake of many other nutritious foods has either stagnated (e.g., whole fruit, fish/shellfish, whole grains) or declined (e.g., **dark green and orange vegetables** and fluid milk) over time.”

The authors apologize to the readers for any inconvenience caused by this modification. The original manuscript will remain online on the article webpage with a reference to this correction.

## Figures and Tables

**Figure 1:**
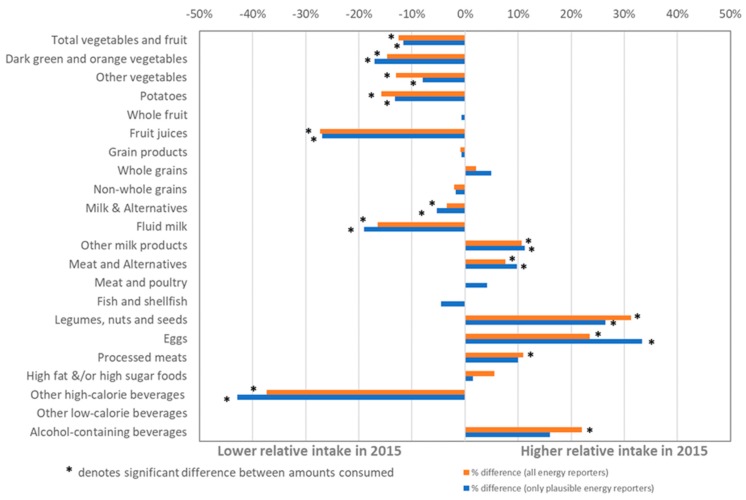
Relative percent (%) change in the mean daily intakes of foods among Canadians age 2 years and older^1^ between 2004 and 2015. From 2004 to 2015, Canadians reported consuming, on average, approximately **15 and 17% fewer dark green and orange vegetables** among plausible reporters and among all energy reporters, respectively. Over the same time period, Canadians reported consuming 8% and 13% fewer “other” vegetables among plausible reporters and among all energy reporters, respectively. Average intake from alcohol-containing beverages estimated for respondents aged 13 years and older only.

**Table 1:** Difference in covariate-adjusted mean daily amounts of vegetable and fruit reported between 2004 and 2015 among Canadians ≥2 years old (all energy reporters and only plausible energy reporters).

	All Energy Reporters						Only Plausible Energy Reporters ^1^					
	Survey Cycle						Survey Cycle					
	2004		2015		Difference		2004		2015		Difference	
	*n* = 32,890		*n* = 18,447				*n* = 12,602		*n* = 7833			
	Mean	SE	Mean	SE	Mean	95% CI	Mean	SE	Mean	SE	Mean	95% CI
**Total vegetables and fruit, servings**												
Young children, 2–5 years	4.2	0.1	4.2	0.0	0.0	−0.3, 0.3	4.3	0.1	3.9	0.1	−0.3	−0.7, 0.1
Children, 6–12 years	4.5	0.1	4.3	0.1	−0.1	−0.4, 0.1	4.4	0.1	4.5	0.1	0.1	−0.3, 0.4
Adolescents, 13–17 years	4.9	0.1	4.5	0.1	−0.4 *	−0.7, −0.0	4.9	0.1	4.5	0.2	−0.5	−0.9, 0.0
Adults, 18–54 years	5.4	0.1	4.6	0.1	−0.8 *	−1.0, −0.6	5.6	0.1	4.7	0.1	−0.9 *	−1.2, −0.6
Older adults, ≥55 years	5.5	0.1	4.7	0.1	−0.8 *	−1.0, −0.6	5.7	0.1	5.1	0.1	−0.6 *	−0.9, −0.3
All ages	5.2	0.0	4.6	0.0	−0.7 *^,†^	−0.8, −0.5	5.4	0.1	4.7	0.1	−0.6 *^,†^	−0.8, −0.4
**Dark green and orange vegetables, servings** ^2^												
**Young children, 2–5 years**	**0.4**	**0.0**	**0.4**	**0.0**	**0.1**	**−0.1, 0.2**	**0.4**	**0.0**	**0.3**	**0.0**	**−0.0**	**−0.1, 0.1**
**Children, 6–12 years**	**0.5**	**0.0**	**0.4**	**0.0**	−0.1 *	**−0.1, −0.0**	**0.4**	**0.0**	**0.4**	**0.0**	**−0.0**	**−0.1, 0.1**
**Adolescents, 13–17 years**	**0.6**	**0.0**	**0.5**	**0.0**	**−0.1**	**−0.1, 0.0**	**0.5**	**0.0**	**0.5**	**0.0**	**−0.0**	**−0.1, 0.1**
**Adults, 18–54 years**	**0.9**	**0.0**	**0.8**	**0.0**	−0.1 *	**−0.2, −0.0**	**0.9**	**0.0**	**0.7**	**0.0**	−0.2 *	**−0.3, −0.0**
**Older adults, ≥55 years**	**1.0**	**0.0**	**0.7**	**0.0**	−0.2 *	**−0.3, −0.1**	**1.0**	**0.0**	**0.8**	**0.0**	−0.2 *	**−0.3, −0.1**
**All ages**	**0.8**	**0.0**	**0.7**	**0.0**	−0.1 *^,†^	**−0.2, −0.1**	**0.8**	**0.0**	**0.7**	**0.0**	−0.1 *^,†^	**−0.2, −0.1**
**“Other” vegetables, servings** ^2^												
**Young children, 2–5 years**	**0.7**	**0.0**	**0.7**	**0.0**	**0.0**	**−0.1, 0.1**	**0.6**	**0.1**	**0.7**	**0.1**	**0.1**	**−0.1, 0.2**
**Children, 6–12 years**	**0.9**	**0.0**	**0.9**	**0.0**	**0.1**	**−0.0, 0.2**	**0.8**	**0.0**	**1.0**	**0.1**	**0.2**	**0.1, 0.4**
**Adolescents, 13–17 years**	**1.1**	**0.0**	**1.0**	**0.0**	**−0.1**	**−0.2, 0.0**	**1.1**	**0.1**	**1.0**	**0.1**	**−0.1**	**−0.3, 0.1**
**Adults, 18–54 years**	**1.6**	**0.0**	**1.4**	**0.0**	−0.2 *	**−0.3, −0.1**	**1.6**	**0.1**	**1.4**	**0.1**	−0.2 *	**−0.4, −0.1**
**Older adults, ≥55 years**	**1.6**	**0.0**	**1.3**	**0.0**	−0.2 *	**−0.4, −0.1**	**1.6**	**0.1**	**1.5**	**0.1**	**−0.1**	**−0.3, 0.0**
**All ages**	**1.5**	**0.0**	**1.3**	**0.0**	−0.2 *^,†^	**−0.3, −0.1**	**1.4**	**0.0**	**1.3**	**0.0**	−0.1 *^,†^	**−0.2, −0.0**
**Potatoes, servings**												
Young children, 2–5 years	0.4	0.0	0.3	0.0	−0.1 *	−0.2, −0.0	0.4	0.0	0.3	0.0	−0.1	−0.2, 0.0
Children, 6–12 years	0.7	0.0	0.5	0.0	−0.2 *	−0.2, −0.1	0.7	0.0	0.5	0.0	−0.2 *	−0.3, −0.1
Adolescents, 13–17 years	0.9	0.0	0.7	0.1	−0.2 *	−0.3, −0.1	0.8	0.0	0.7	0.1	−0.2 *	−0.3, −0.0
Adults, 18–54 years	0.8	0.0	0.6	0.0	−0.2 *	−0.2, −0.1	0.8	0.0	0.6	0.0	−0.1 *	−0.2, −0.0
Older adults, ≥55 years	0.8	0.0	0.8	0.0	0.0	−0.1, 0.1	0.8	0.0	0.8	0.0	0.0	−0.1, 0.2
All ages	0.8	0.0	0.6	0.0	−0.1 *	−0.2, −0.1	0.8	0.0	0.7	0.0	−0.1 *	−0.2, −0.0
**Whole fruit, servings**												
Young children, 2–5 years	1.3	0.1	1.8	0.1	0.4 *	0.2, 0.6	1.5	0.1	1.6	0.1	0.2	−0.1, 0.5
Children, 6–12 years	1.3	0.1	1.5	0.1	0.2 *	0.0, 0.3	1.4	0.1	1.5	0.1	0.2	−0.1, 0.4
Adolescents, 13–17 years	1.0	0.4	1.3	0.1	0.2 *	0.0, 0.4	1.0	0.1	1.3	0.1	0.2 *	0.0, 0.5
Adults, 18–54 years	1.3	0.0	1.2	0.0	−0.1	−0.2, 0.1	1.4	0.1	1.3	0.1	−0.1	−0.3, 0.1
Older adults, ≥55 years	1.5	0.0	1.4	0.0	−0.1	−0.2, 0.0	1.5	0.1	1.5	0.1	−0.1	−0.2, 0.1
All ages	1.3	0.0	1.3	0.0	0.0 ^†^	−0.1, 0.1	1.4	0.1	1.4	0.0	0.0 ^†^	−0.1, 0.1
**Fruit juices, servings**												
Young children, 2–5 years	1.4	0.1	1.0	0.1	−0.4 *	−0.6, −0.2	1.4	0.1	0.9	0.1	−0.4 *	−0.7, −0.2
Children, 6–12 years	1.2	0.0	1.0	0.0	−0.1 *	−0.3, −0.0	1.2	0.1	1.0	0.1	−0.1	−0.3, 0.0
Adolescents, 13–17 years	1.3	0.1	1.0	0.1	−0.3 *	−0.5, −0.1	1.5	0.1	1.1	0.1	−0.4 *	−0.7, −0.1
Adults, 18–54 years	0.8	0.0	0.6	0.0	−0.2 *	−0.3, −0.2	0.9	0.1	0.6	0.1	−0.2 *	−0.4, −0.1
Older adults, ≥55 years	0.7	0.0	0.4	0.0	−0.2 *	−0.3, −0.1	0.7	0.0	0.5	0.0	−0.3 *	−0.4, −0.2
All ages	0.9	0.0	0.6	0.0	−0.2 *^,†^	−0.3, −0.2	0.9	0.0	0.7	0.0	−0.2 *	−0.3, −0.2

CI, Confidence intervals. SE, Standard error. Data were weighted for the Canadian population, but unweighted sample sizes are shown. ^1^ Children and adults with measured weight and height were classified as either under-, plausible or over-energy reporters based on the ratio of reported vs. total energy expenditure (TEE). TEE was based on equations that account for height, weight, age, sex, physical activity levels, and weight status (normal weight vs. overweight/obese) [2]. Physical activity levels were assumed to be low for children aged 13 and younger and sedentary for respondents aged 14 and older. ^2^ The revised values for mean amounts of dark green and orange vegetables and “other” vegetables for 2004 and 2015, as well as the corrected mean difference over time are highlighted in **bold**. * Significant difference between 2004 and 2015 was tested using multivariable linear regression models adjusted for daily energy intake, age in years, ethnicity, immigrant status, household-level education, smoking status, and supplement use. Sample sizes differ slightly from the unadjusted models due to missing data for ethnicity, immigrant status, household-level education, and supplement use. Due to large numbers of missing data for the smoking variable (“not applicable” for respondents under 12 years, “refused”, “don’t know” or “not stated”), a dummy variable was created to avoid dropping these respondents in covariate-adjusted linear models. ^†^ *p*-value from the Wald test testing the joint significance of adding the age group and survey year interaction product terms is significant (*p*-value < 0.05).
